# Response-Patch CNN–Transformer Multitask Learning Model for VOCs Identification Using Electronic Nose

**DOI:** 10.3390/s26216961

**Published:** 2026-11-04

**Authors:** Junhui Xia, Ning Zhang, Mingdong Chen, Haixia Mei

**Affiliations:** Key Lab Intelligent Rehabil & Barrier Free Disable (Ministry of Education), Changchun University, Changchun 130022, China

**Keywords:** electronic nose, response-patch sequences, CNN–Transformer, multitask learning, volatile organic compounds (VOCs)

## Abstract

**Highlights:**

**What are the main findings?**

- The proposed CNN–Transformer multitask model accurately identifies VOCs and predicts their concentrations.
- Response-patch representation and task-specific feature learning jointly enhance VOC detection performance and model robustness.

**What are the implications of the main findings?**

- This study provides an integrated electronic-nose solution for simultaneous classification and concentration prediction of VOCs by effectively combining local response dynamics with long-range temporal dependencies.
- The effectiveness of response-patch representation and task-specific feature enhancement demonstrates the model’s potential for other multichannel VOC sensing and time-series analysis tasks.

**Abstract:**

The electronic nose system is susceptible to cross-sensitivity, noise, and baseline drift, making simultaneous qualitative identification and quantitative prediction of volatile organic compounds (VOCs) challenging. To address these issues, this study proposes a CNN–Transformer multitask learning model based on response-patch sequences. A multichannel activity curve is first constructed to locate response onset and extract fixed-length samples. The long response sequence is then divided into ordered patches using a sliding window. A CNN captures local dynamic patterns associated with signal rise, peak, and recovery, while learnable positional embeddings and a Transformer model the sequential order and long-range dependencies among patches. Task-specific squeeze-and-excitation attention branches adaptively enhance classification- and regression-related features, and an uncertainty-weighted loss balances the two learning objectives. With a patch length of 75 and a stride of 37, the model achieved an Accuracy of 0.9717, an F1-score of 0.9696, an MAE of 5.0789 ppm, and an R^2^ of 0.9467. Comparative and ablation experiments confirmed its superior overall performance and the effectiveness of the proposed modules. The method provides a feasible solution for the integrated identification and quantitative analysis of VOCs in electronic-nose applications.

## 1. Introduction

Volatile organic compounds (VOCs) are widely present in industrial production, environmental monitoring, food safety, and medical diagnosis. Some VOCs are toxic and pose potential health risks. Therefore, their rapid and accurate detection is of considerable importance [1,2]. Conventional analytical methods, including gas chromatography, mass spectrometry, and gas chromatography–mass spectrometry, provide high detection accuracy but are limited by expensive equipment, complicated operation procedures, and limited suitability for on-site applications. In comparison, electronic noses based on gas-sensor arrays offer advantages such as rapid response, low cost, and easy integration, providing an effective approach for VOC detection [3,4].

Early electronic-nose data analysis mainly relied on traditional machine-learning methods, such as k-nearest neighbors (KNN), support vector machines (SVM), and random forests (RF), whose performance primarily depended on handcrafted feature design [5]. With the development of deep learning, convolutional neural networks (CNNs), recurrent neural networks (RNNs), long short-term memory networks (LSTMs), and temporal convolutional networks (TCNs) have gradually been applied to gas-response time-series modeling to extract local dynamic features and temporal dependencies at different scales [6,7,8]. Transformers further enhance global dependency modeling through self-attention mechanisms [9], while multitask learning has increasingly been used to simultaneously perform gas classification and concentration prediction [10].

In recent years, electronic-nose research has gradually shifted from individual gas classification toward the joint modeling of gas-species identification and concentration prediction. Wang et al. developed a multitask deep-learning model that simultaneously performed gas classification, concentration prediction, and state assessment, achieving a classification accuracy of 95.2% for 12 VOCs [11]. Subsequent studies have explored joint prediction using traditional machine-learning [12], Transformer-based multitask modeling [13], time–frequency feature fusion [14], and other feature-fusion strategies [15,16,17]. As the complexity of target gases increases, multitask methods have also been extended to the analysis of VOC mixtures. Mei et al. proposed MTL-RCANet for binary VOC mixtures, combining feature fusion with dynamic loss weighting to achieve gas identification and concentration prediction. The model achieved a classification accuracy of 94.86% and a concentration-prediction R^2^ of 0.95 [18]. Multitask learning has also been applied to the rapid classification and concentration prediction of VOCs [19]. Overall, qualitative and quantitative electronic-nose analysis is evolving from separate single-task models toward multitask joint modeling and from individual gas detection toward more complex scenarios involving gas mixtures.

Recent efforts to enhance the representation of gas response time-series features have shifted from local feature extraction toward joint modeling of local dynamics and long-range temporal information. Wu et al. proposed TETCN, which combines a Transformer encoder with a TCN to extract contextual information and long-range temporal features, achieving a classification accuracy of 99.8% [20]. Jiang et al. developed TFA-CNN, which integrates time and frequency domain features through time–frequency attention, achieving a classification accuracy of 100%, a concentration-prediction mean absolute error (MAE) of 1.542 ppm, and an R^2^ of 0.990 [14]. Yang et al. proposed MCCT-Net, which combines multiscale convolution with a Transformer to jointly extract local and global features, achieving classification accuracies of 98.67% and 98.52% on two electronic-nose datasets [21]. Meanwhile, temporal attention mechanisms [22], lightweight hybrid networks [23], and Transformer–ResNet1D architectures [24] have also been applied to gas concentration prediction and joint classification–regression modeling. These studies demonstrate that the joint modeling of local and global information has become an important direction for improving the representation of electronic-nose time-series signals.

For long-duration response sequences, effective organization of dynamic information from different response stages has attracted growing attention. Fu et al. used multi-interval data sampling to exploit information from different response stages and improve real-time gas-recognition performance [25]. In general time-series modeling, patch-based Transformer frameworks divide long time series into ordered local patches to improve the representation of local temporal patterns and long-range dependencies, providing a useful reference for organizing long electronic-nose response sequences [26]. In addition, research on low-power electronic noses for real-time gas recognition has further facilitated the effective use of long-term response information [27]. Dynamic response fragments combined with multitask learning provide a practical way to exploit informative temporal patterns from different stages of gas-response signals [28].

Multitask learning also introduces the challenge of balancing shared and task-specific feature representations. Ma et al. incorporated an SE-ResNet module into an electronic-nose gas-classification network to enhance channel-wise feature representation and feature extraction [29]. Vandenhende et al. noted that different tasks have distinct feature requirements and optimization objectives [30]. Therefore, shared and task-specific representations must be appropriately coordinated. Recent studies have further expanded intelligent electronic-nose detection toward lightweight gas classification and concentration prediction, single-sensor multitask VOC identification, quantitative analysis of gas mixtures, and adaptive multigas monitoring [31,32,33]. Consequently, retaining task-specific features while effectively utilizing shared information has become an important issue in multitask modeling.

Despite advances in joint qualitative-quantitative detection, time-series feature modeling, and multitask learning, few studies have fully exploited both local dynamic features inside response patches and long-range temporal dependencies across patches. The influence of different response-patch scales on local and global feature modeling has also not been systematically investigated. Moreover, classification and regression tasks focus on different aspects of feature information, and a fully shared representation may limit the expression of task-specific information.

To address these limitations, this study proposes a CNN–Transformer multitask learning model based on response-patch sequence modeling. The model employs a CNN to extract local dynamic features within each response patch and uses positional embeddings and a Transformer to model long-range temporal dependencies among patches. A task-specific SE mechanism is further introduced to adaptively recalibrate the features required for classification and regression, thereby enabling the joint modeling of VOC-species identification and concentration prediction.

## 2. Materials and Methods

### 2.1. Data Acquisition

The data were collected using eight MQ-series metal oxide semiconductor (MOS) gas sensors(Zhengzhou Winsen Electronics Technology Co., Ltd., Zhengzhou, China), labeled Sensor 1–8. Their models and nominal target gases are listed in Table 1. Differing cross-sensitivities generated multichannel VOC response patterns.

Supplementary Figure S1a shows the dual-chamber system with separate sensor array and data acquisition boards. A microsyringe pump delivered liquid onto a heating stage set approximately 5 °C below each compound’s boiling point. Injection volumes were calculated from molecular weight, liquid density, and target concentration. Fans operating under closed-loop control regulated airflow. Signals were sampled at 2 Hz and transmitted to the host via 433 MHz wireless communication.

The dataset included responses to 12 VOCs: acetone, ethanol, ethylene glycol, n-propanol, isopropanol, methanol, butyl acetate, ethyl acetate, toluene, n-hexane, xylene, and benzaldehyde. As shown in Figure S1b, each run began with 5 min in air for baseline tracking, followed by 5min exposures at concentrations increasing from 10 to 100 ppm in 10 ppm increments. The chamber door was opened for air recovery, lasting 5 min between exposures and 10 min after the final 100 ppm exposure [11,28]. Figure S1c marks the peak within the exposure phase and illustrates recovery toward baseline. Five complete runs per VOC were selected, yielding 600 response samples.

Measurements were conducted in air at atmospheric pressure, with room temperature and relative humidity of (26 ± 2) °C and (50 ± 10)%, respectively. Both were monitored and recorded using chamber sensors.

**Figure 1:**
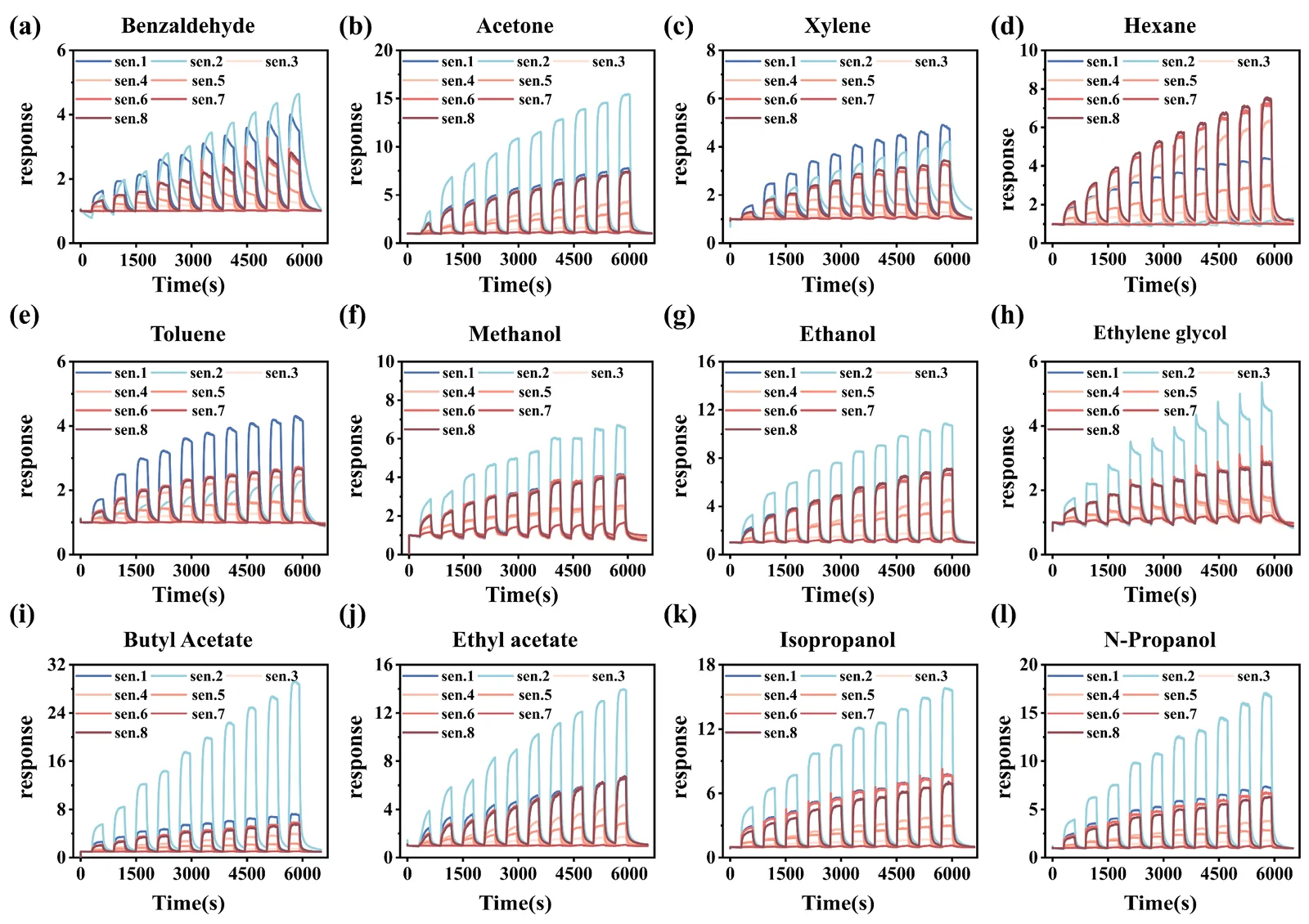
Time–response curves of the gas sensor array: (**a**) Benzaldehyde; (**b**) Acetone; (**c**) Xylene; (**d**) Hexane; (**e**) Toluene; (**f**) Methanol; (**g**) Ethanol; (**h**) Ethylene glycol; (**i**) Butyl acetate; (**j**) Ethyl acetate; (**k**) Isopropanol; and (**l**) N-propanol.

The time–response curves of the gas-sensor array obtained during the experiments are shown in Figure 1. Additional response curves of individual sensors to the 12 VOCs are provided in Supplementary Figure S2.

In Figure 1, panels (a)–(l) show the sensor response curves for benzaldehyde, acetone, xylene, hexane, toluene, methanol, ethanol, ethylene glycol, butyl acetate, ethyl acetate, isopropanol, and n-propanol, respectively. Figure S2a–h further presents the responses of Sensors 1–8 to all 12 VOCs over the concentration range of 10–100 ppm. Individual sensors responded to multiple VOCs, but their response magnitudes and temporal patterns varied across different gases. These differences reflect the cross-sensitivity and complementary response characteristics of the sensor array, providing discriminative multichannel information for subsequent VOC identification. The gas response value was calculated using the following equation:

(1) $$S=\frac{R_{0}}{R}$$

In this equation, *R*_0_ denotes the resistance of the gas sensor in air, and *R* denotes its resistance when exposed to the target gas. The calculated response reflects the change in sensor resistance induced by the target gas relative to the baseline resistance in air.

### 2.2. Data Preprocessing

**Figure 2:**
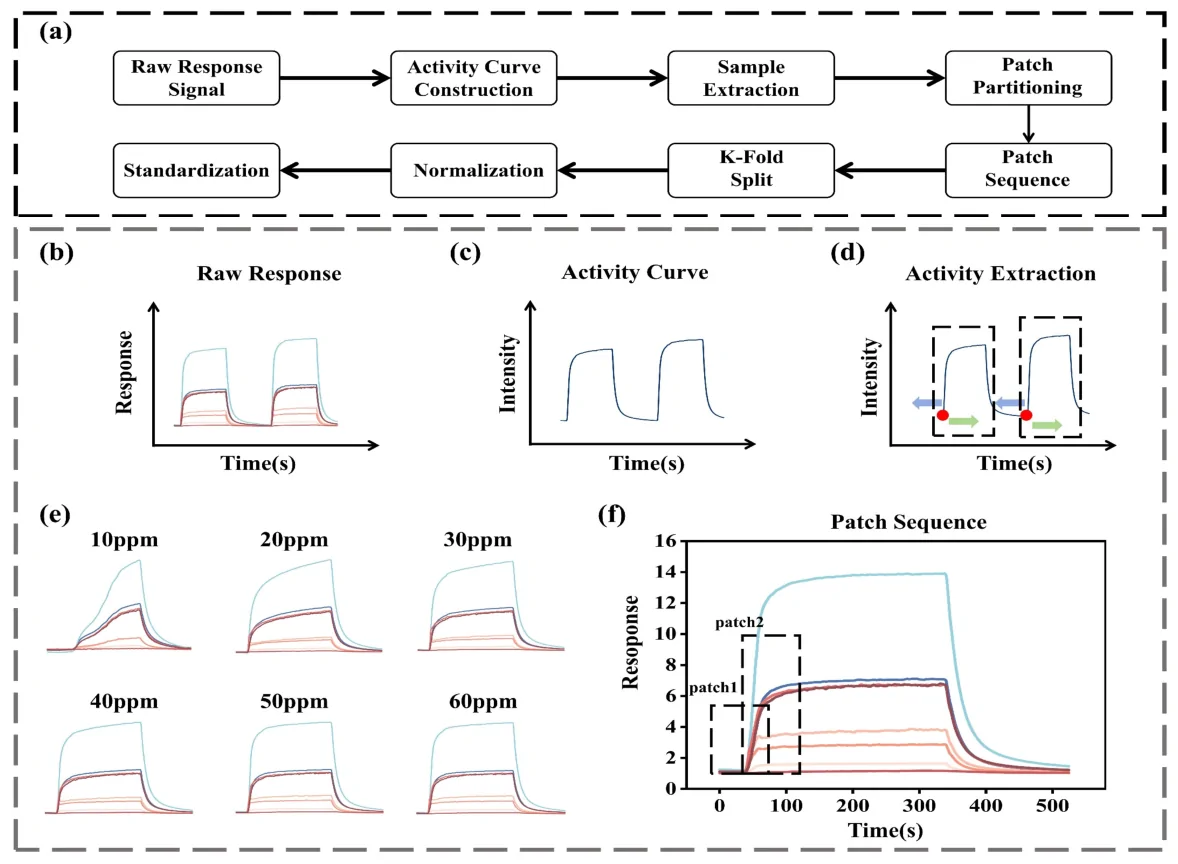
Data preprocessing and response-patch sequence construction: (**a**) Overall workflow. (**b**) Raw multichannel responses. (**c**) Activity-curve construction. (**d**) Response-onset detection and sample extraction. (**e**) Fixed-length samples at 10–60 ppm. (**f**) Response-patch sequence construction.

As shown in Figure 2, the data preprocessing workflow is summarized in Figure 2a. The raw multichannel gas-response signals in Figure 2b were used to construct the activity curve in Figure 2c. The response onset of each concentration stage was identified using activity intensity, local slope, and the number of activated channels, followed by fixed-length sample extraction, as illustrated in Figure 2d. Representative extracted samples at concentrations of 10–60 ppm are shown in Figure 2e. Each extracted sample was then divided into overlapping patches and arranged chronologically to form a response-patch sequence, as illustrated in Figure 2f.

#### 2.2.1. Activity-Curve Construction

Before model training, independent samples corresponding to each concentration stage were extracted from the continuously acquired multichannel gas-response signals. Because each experiment contained multiple concentration stages and their associated response and recovery processes, directly using the complete continuous curves could result in information mixing between stages. Therefore, an activity curve was constructed by combining prior knowledge of the experimental procedure with multichannel response characteristics. The actual response onset of each concentration stage was then located, and a fixed-length response sample was extracted accordingly.

The first 100 sampling points, corresponding to approximately 50 s, were selected from the initial stage as the baseline reference segment. This length reduced the influence of instantaneous noise on the estimation of baseline statistics while avoiding slow drift or environmental changes caused by an excessively long window. The sensor array contained eight response channels indexed by *c*, where *c* = 1,2,…,8. At sampling point *t*, the response of channel *c* was denoted by *x_c_*(*t*). The baseline mean *μ_c_* and standard deviation *σ_c_* of each channel were calculated from the baseline reference segment as follows:

(2) $$\mu_{c}=\frac{1}{N_{b}}\sum_{t=1}^{N_{b}}x_{c}(t)$$

(3) $$\sigma_{c}=\sqrt{\frac{1}{N_{b}}\sum_{t=1}^{N_{b}}{\left(x_{c}(t)-\mu_{c}\right)}^{2}}$$

where *N_b_* denotes the number of sampling points in the baseline reference segment and was set to 100. *μ_c_* and *σ_c_* denote the baseline mean and standard deviation of sensor channel *c*, respectively. Calculating the baseline statistics for each channel reduces the influence of channel-to-channel differences in initial response levels and fluctuation amplitudes on activity-curve construction.

Subsequently, the response data from each sensor channel were standardized using the corresponding baseline statistics, enabling the response variations in different channels to be compared on a relatively consistent scale. The standardized response of the c-th sensor channel at sampling point *t* was expressed as follows:

(4) $$z_{c}(t)=\frac{x_{c}(t)-\mu_{c}}{\sigma_{c}}$$

After standardizing each channel, the activity intensity *A*(*t*) was calculated as the mean absolute standardized response across the eight channels at each sampling point, thereby preventing cancellation caused by differences in response direction:

(5) $$A(t)=\frac{1}{C}\sum_{c=1}^{C}\left|z_{c}(t)\right|$$

where *A*(*t*) denotes the multichannel activity intensity at sampling point *t*, reflecting the overall deviation of the sensor array from its baseline state.

The activity curve was smoothed, and its local slope was calculated. Near the theoretical onset of each concentration stage, the actual response onset was identified by jointly considering the activity intensity, local slope, and number of activated channels, thereby reducing the influence of single-channel noise, abnormal fluctuations, and peak shifts on sample extraction.

After determining the response onset, the signals from all eight sensor channels were synchronously extracted using this position as the temporal anchor. Each sample consisted of an 80-point pre-response baseline segment, a 600-point gas-exposure segment, and a 370-point air-recovery segment, giving a total length of

(6) $$L_{\mathrm{total}}=L_{\mathrm{pre}}+L_{\mathrm{res}}+L_{\mathrm{rec}}=80+600+370=1050$$

where *L_pre_*, *L_res_*, and *L_rec_* denote the lengths of the pre-response baseline, gas-exposure, and air-recovery segments, respectively. Based on the experimental procedure and response characteristics, the 80-point baseline segment corresponded to approximately 40 s, the 600-point exposure segment covered the complete 5 min gas-exposure period, and the 370-point recovery segment corresponded to approximately 185 s, capturing the main recovery process while reducing redundant late-stage information. The baseline standardization described above was used only to construct the activity curve and locate the response onset, whereas the extracted raw responses from the eight sensor channels were used as the model input.

#### 2.2.2. Response Segment Partitioning and Sequence Construction

Each extracted sample was represented as a 1050 × 8 two-dimensional response matrix, corresponding to 1050 time points and eight sensor channels, respectively. A sliding window of length L and stride S was applied along the temporal dimension to divide each sample into consecutive segments. Let *x_i_*_,_*_t_* denote the eight-channel response vector of the *i*-th sample at time *t*. Then, its *j*-th response segment can be expressed as

(7) $$X_{i,j}^{\mathrm{seg}}=\left[x_{i,t_{j}},x_{i,t_{j+1}},\ldots ,x_{i,t_{j+L-1}}\right]$$

In this expression, *t_j_* denotes the starting position of the j-th segment, and $X_{i,j}^{\mathrm{seg}}$ represents a response segment containing *L* consecutive sampling points with a size of *L* × 8. During segmentation, all eight channels remain synchronized. The window moves chronologically with a stride of S, while adjacent segments retain a certain degree of overlap to preserve the complete multichannel response information within each time interval.

To ensure complete coverage of the response sequence, fixed-stride segmentation was applied when 1050 L was divisible by S. Otherwise, an additional segment aligned with the end of the sequence was appended after regular segmentation to prevent the loss of tail information.

The resulting P response segments were arranged in their original temporal order to form a response-segment sequence of size P × L × 8, where L denotes the segment length. Each segment preserved local dynamic information, while the temporal relationships between segments were maintained. In this study, L ranged from 55 to 195 sampling points in increments of 20, and the sliding stride was set to half of the segment length and rounded down to the nearest integer. Through this procedure, each complete response was converted into an ordered segment sequence, providing the input for the CNN to extract local intra-segment features and for the Transformer to model global inter-segment temporal dependencies.

#### 2.2.3. Data Partitioning and Standardization

Five-fold cross-validation grouped by the original experimental files was employed to prevent data leakage caused by samples from the same source appearing in both the training and validation sets. The response-segment sequences were partitioned at the complete-sample level, with all segments from the same sample assigned to the same subset. Each fold contained 480 training samples and 120 validation samples, and the validation set covered all gas classes and concentration levels to evaluate the model’s cross-file generalization ability.

To reduce differences in response amplitude across sensor channels, the minimum and maximum values of each channel were calculated using only the training set of the current fold, followed by Min–Max normalization:

(8) $$x^{\prime }=\frac{x-x_{\min}}{x_{\max}-x_{\min}}$$

Here, *x* and *x*′ denote the original and normalized response values, respectively, while *x*_min_ and *x*_max_ are the minimum and maximum values of the corresponding channel in the training set of the current fold. The validation set was transformed using only the parameters derived from the training set to prevent information leakage.

For the concentration regression task, concentration labels ranging from 10 to 100 ppm were standardized using the Z-score method to reduce the influence of label scale on the regression loss and parameter updates, thereby improving the stability of multitask training:

(9) $$y^{\prime }=\frac{y-\mu_{y}}{\sigma_{y}}$$

Here, *y* and *y*′ denote the original and standardized concentration labels, respectively, while *μ_y_* and *σ_y_* are the mean and standard deviation calculated from the concentration labels in the current training fold. The model was trained using the standardized labels, and the predictions were transformed back to the original ppm scale for evaluation. All standardization parameters were derived solely from the training set.

### 2.3. CNN–Transformer Multitask Learning Model

**Figure 3:**
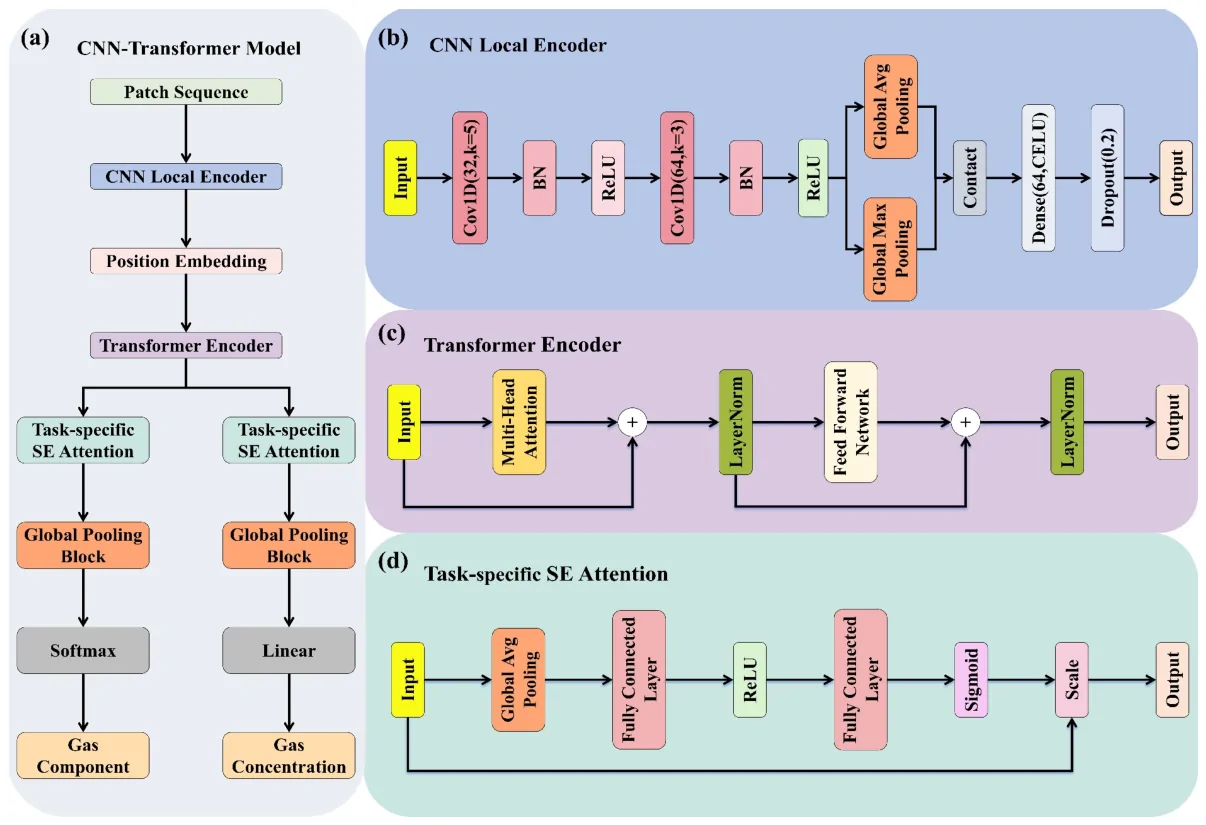
Architecture of the CNN–Transformer multitask learning model. (**a**) Overall model framework. (**b**) CNN-based local feature encoder. (**c**) Transformer-based global temporal encoder. (**d**) task-specific SE attention mechanism.

The proposed CNN–Transformer multitask model consists of a CNN-based local feature encoder, positional embeddings, a Transformer-based global temporal encoder, task-specific SE attention modules, and two task-specific output branches, as shown in Figure 3.

The CNN extracts local dynamic features within each segment, positional embeddings introduce temporal information, and the Transformer models long-range dependencies across segments. Task-specific SE modules further enhance classification- and regression-related features, which are passed through global pooling and then processed by a Softmax classification head and a linear regression head for gas identification and concentration prediction, respectively. This design enables joint modeling of local, global, and task-specific information. Although the regression head employs a linear output layer, the overall network can model nonlinear relationships between sensor responses and gas concentrations through the nonlinear feature transformations performed by the CNN, Transformer, and task-specific SE modules. The linear output layer maps the learned features to continuous concentration values without imposing an explicit constraint on the prediction range.

#### 2.3.1. CNN-Based Local Feature Encoder

The CNN-based local feature encoder extracts short-term dynamic patterns within each response segment, including response rise, local fluctuations, peak variations, and recovery decay. As shown in Figure 3b, the encoder consists of two one-dimensional convolutional layers with 32 and 64 kernels of sizes 5 and 3, respectively. Each convolutional layer is followed by batch normalization and ReLU activation. The convolutional features are processed using global average pooling and global max pooling, concatenated, and then mapped to a 64-dimensional feature vector through a fully connected layer with GELU activation and a dropout rate of 0.2. For a sample containing P segments, the CNN produces a feature sequence of size P × 64, which serves as the input to the positional embedding and Transformer modules.

#### 2.3.2. Positional Embedding Module

The 64-dimensional segment features produced by the CNN are arranged in their original temporal order. Because self-attention does not inherently encode explicit positional information whereas gas responses exhibit distinct temporal stages, a 64-dimensional learnable positional embedding is added element-wise to each corresponding segment feature, as shown in Figure 3a. For a sample containing P segments, the resulting feature sequence retains a size of P × 64 and is used as the input to the Transformer encoder.

#### 2.3.3. Transformer-Based Global Temporal Encoder

The position-embedded feature sequence is fed into a single-layer Transformer encoder for global temporal modeling, as shown in Figure 3c. The multi-head self-attention module uses two attention heads to capture long-range dependencies between response segments, while the feed-forward network has a hidden dimension of 128 and employs GELU activation. Residual connections, layer normalization, and dropout with a rate of 0.2 are used to improve training stability and reduce overfitting. The encoded feature sequence retains a size of P × 64 and serves as the shared input to the task-specific SE modules and the two prediction tasks.

#### 2.3.4. Task-Specific Squeeze-and-Excitation Attention Mechanism

Gas classification focuses on discriminative response patterns among different VOCs, whereas concentration regression emphasizes the continuous relationship between response features and concentration. Therefore, as shown in Figure 3d, independent SE modules are introduced after the Transformer for the two tasks. For the shared feature sequence of size P × 64, global average pooling first produces a 64-dimensional feature descriptor. Two fully connected layers then perform dimensional compression and recovery from 64 to 8 and back to 64, followed by a Sigmoid function to generate feature weights. These weights are multiplied element-wise with the original features to achieve task-specific recalibration, enabling the two branches to emphasize features relevant to gas classification and concentration prediction, respectively.

## 3. Results and Discussion

### 3.1. Model Training Settings

To ensure comparability, all experiments used the same training settings except for the variables under investigation. Five-fold cross-validation was performed with a fixed random seed of 42. The model was trained using Adam for up to 200 epochs with a batch size of 8 and an initial learning rate of 0.0005. Learning-rate decay and early stopping were applied. The SE reduction ratio was set to 8. Further details are provided in Table 2.

### 3.2. Effect of Response-Patch Length on Model Performance

To evaluate the effect of response-patch partitioning and length on model performance, eight configurations with patch lengths ranging from 55 to 195 sampling points were investigated. In addition, a single-patch configuration (1050/1050) was included as a reference to assess the effectiveness of partitioning the complete response sequence. The model components, training strategy, and other training hyperparameters were kept unchanged, while the number of patches and associated positional embedding parameters varied with the configuration. All experiments employed identical file-level five-fold cross-validation, and performance was evaluated using Accuracy, F1-score, MAE, and R^2^, as reported in Table 3.

Table 3 shows that model performance varied with the patch configuration. The 75/37 configuration achieved the best overall performance, with an accuracy of 0.9717, an F1-score of 0.9696, an MAE of 5.0789 ppm, and an R^2^ of 0.9467. In comparison, the single-patch configuration (1050/1050) achieved an accuracy of 0.9633, an F1-score of 0.9622, an MAE of 5.7431 ppm, and an R^2^ of 0.9212. This comparison suggests that dividing the complete response sequence into multiple patches facilitates local dynamic feature extraction and inter-patch temporal modeling. Although overlapping patches introduce additional computational overhead compared with the single-patch configuration, the model parameters increased only from 69,149 to 70,877. Shorter patches may fragment local response dynamics, whereas longer patches reduce the number of temporal units. Therefore, the 75/37 configuration provided a suitable balance between predictive performance and model complexity and was adopted for subsequent comparison and ablation experiments.

### 3.3. Overall Performance of the CNN–Transformer Multitask Model

After identifying 75/37 as the optimal response-patch setting, the model was evaluated based on five-fold cross-validation and training convergence. Classification and regression metrics, confusion matrices, and concentration-prediction scatter plots were used to assess VOC identification and concentration prediction, while the training and validation loss curves were analyzed to evaluate model convergence and stability.

#### 3.3.1. Five-Fold Cross-Validation Performance

To evaluate the stability and generalization ability of the CNN–Transformer multitask model, five-fold cross-validation was performed using the 75/37 response-patch setting. The classification and regression results are provided in Table 4.

**Figure 4:**
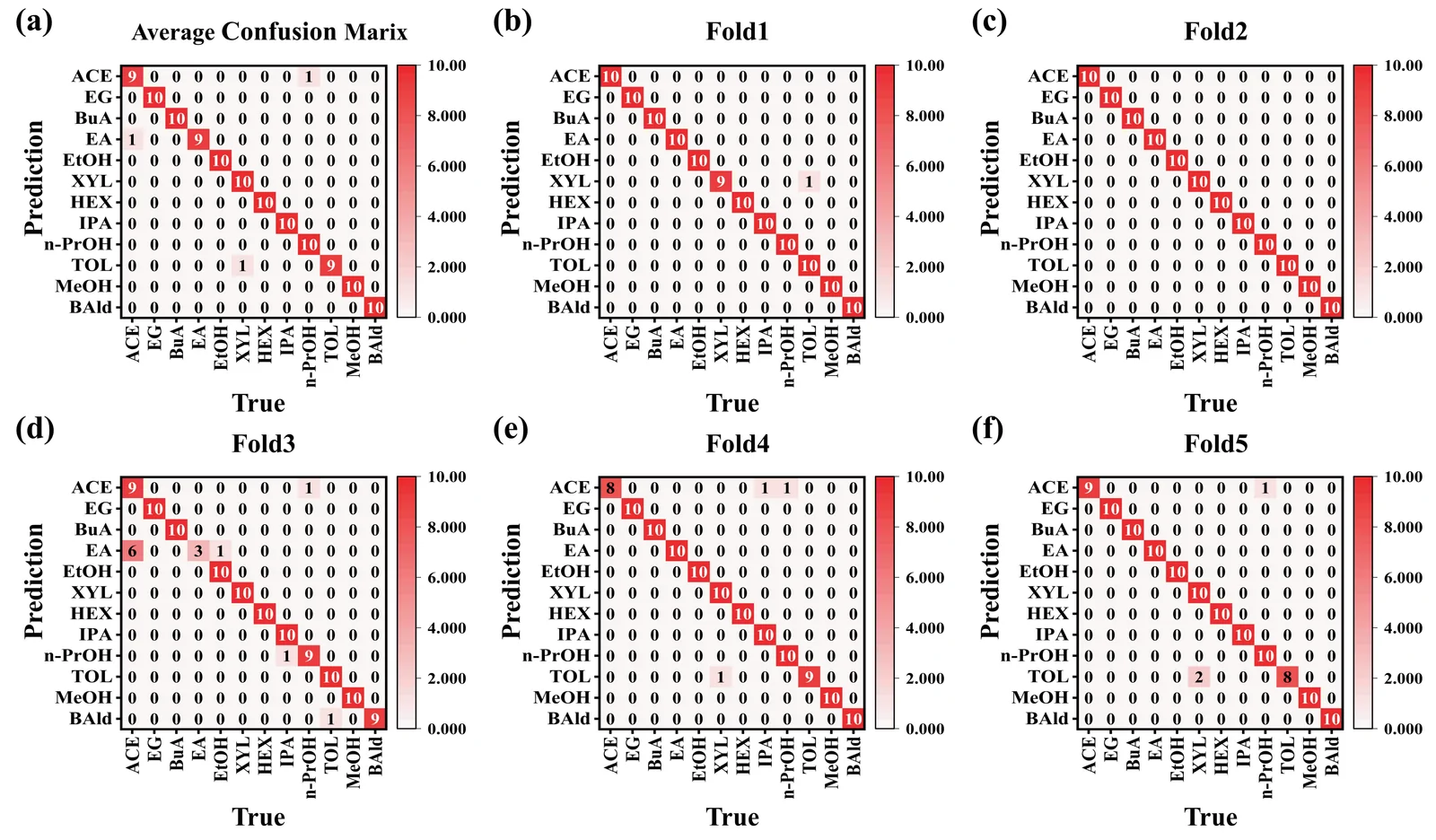
Confusion matrices for gas identification under five-fold cross-validation. (**a**) Rounded mean confusion matrix across the five folds. (**b**–**f**) Confusion matrices for folds 1–5, respectively.

As shown in Table 4, the model achieved a mean Accuracy of 0.9717 and an F1-score of 0.9696 for gas identification, together with an MAE of 5.0789 ppm and an R^2^ of 0.9467 for concentration prediction. The small standard deviations across the evaluation metrics indicate good stability and generalization. Figure 4 presents the confusion matrices from five-fold cross-validation to further illustrate the classification performance for each gas category.

Figure 4 presents the gas identification results obtained from five-fold cross-validation. Figure 4a shows the rounded mean confusion matrix, while Figure 4b–f show the results for the individual folds. Most samples are concentrated along the main diagonal, demonstrating effective identification of the 12 VOCs. The few misclassifications between certain categories may be attributed to similarities in their sensor response patterns.

**Figure 5:**
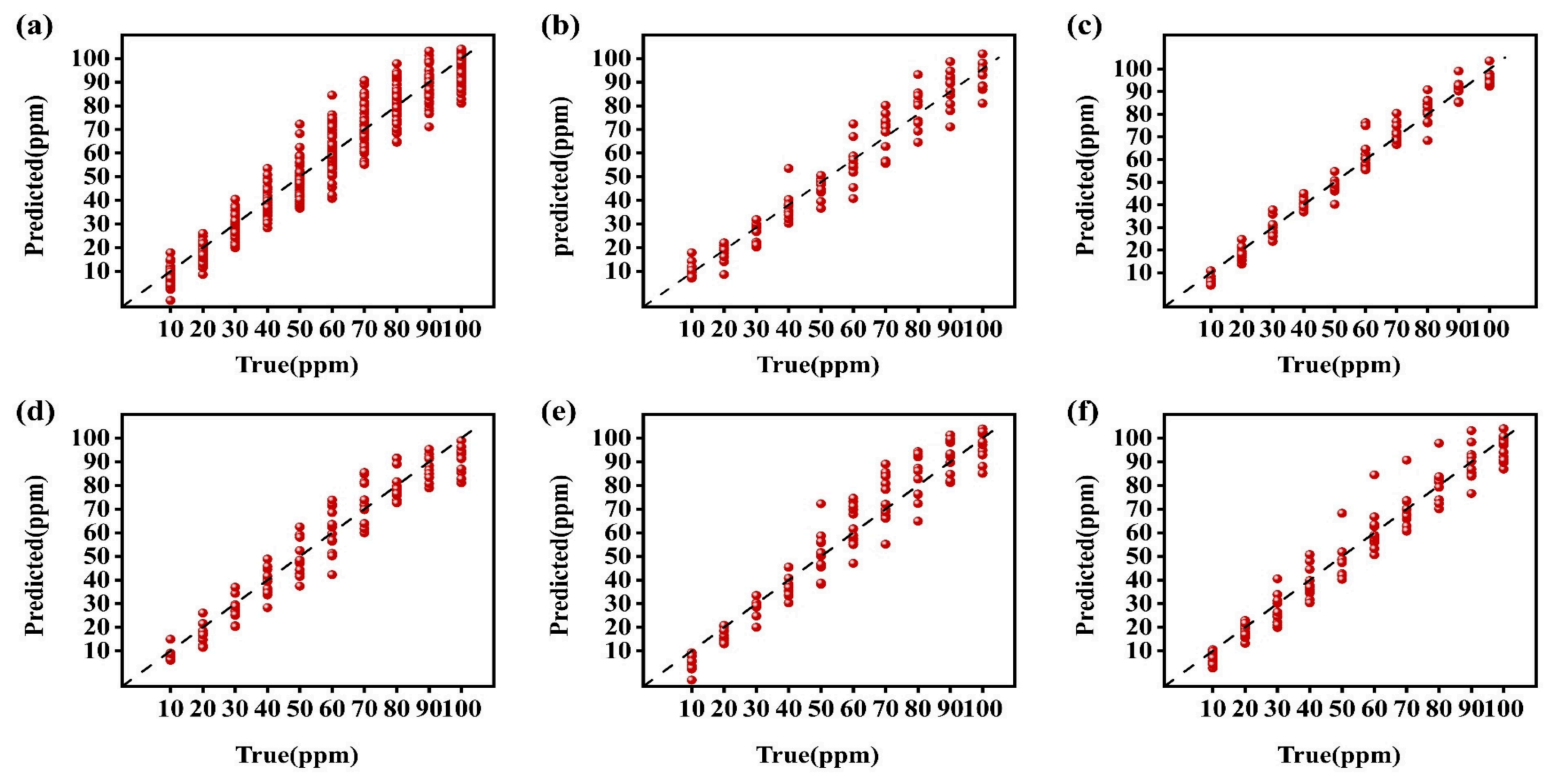
Concentration prediction results for all 12 VOCs under five-fold cross-validation: (**a**) Combined predictions for all 600 validation samples from the five folds. (**b**–**f**) Predictions for the 120 validation samples in folds 1–5, respectively.

To further evaluate concentration prediction, Figure 5 compares the predicted and true concentrations over the range of 10–100 ppm under five-fold cross-validation, allowing assessment of the overall fit, prediction bias at different concentration levels, and stability across folds.

Figure 5 presents the concentration prediction results for all 12 VOCs under five-fold cross-validation, comprising 600 validation samples. Predictions were transformed back to ppm using the mean and standard deviation of the corresponding training fold. Most predictions were close to the identity line, indicating good overall performance across 10–100 ppm. For low (10–30 ppm), medium (40–70 ppm), and high (80–100 ppm) concentration ranges, the MAEs were 3.51, 5.66, and 5.87 ppm, with corresponding mean biases of −2.33, −0.43, and −1.67 ppm. These results indicate some underestimation, particularly at lower concentrations, potentially associated with sensor noise, baseline drift, and inter-run response variations.

Overall, the CNN–Transformer multitask model demonstrated favorable VOC identification and concentration prediction performance through the integration of local dynamic and global temporal features. The following sections further examine model convergence and the contributions of individual components.

#### 3.3.2. Loss Function

To balance the joint optimization of gas classification and concentration regression, a learnable uncertainty-based weighting strategy was adopted to construct the multitask loss function [34], which is defined as follows:

(10) $${\mathrm{Loss}}_{\mathrm{total}}=e^{-s_{\mathrm{cls}}}{\mathrm{Loss}}_{\mathrm{cls}}+e^{-s_{\mathrm{reg}}}{\mathrm{Loss}}_{\mathrm{reg}}+s_{\mathrm{cls}}+s_{\mathrm{reg}}$$

**Figure 6:**
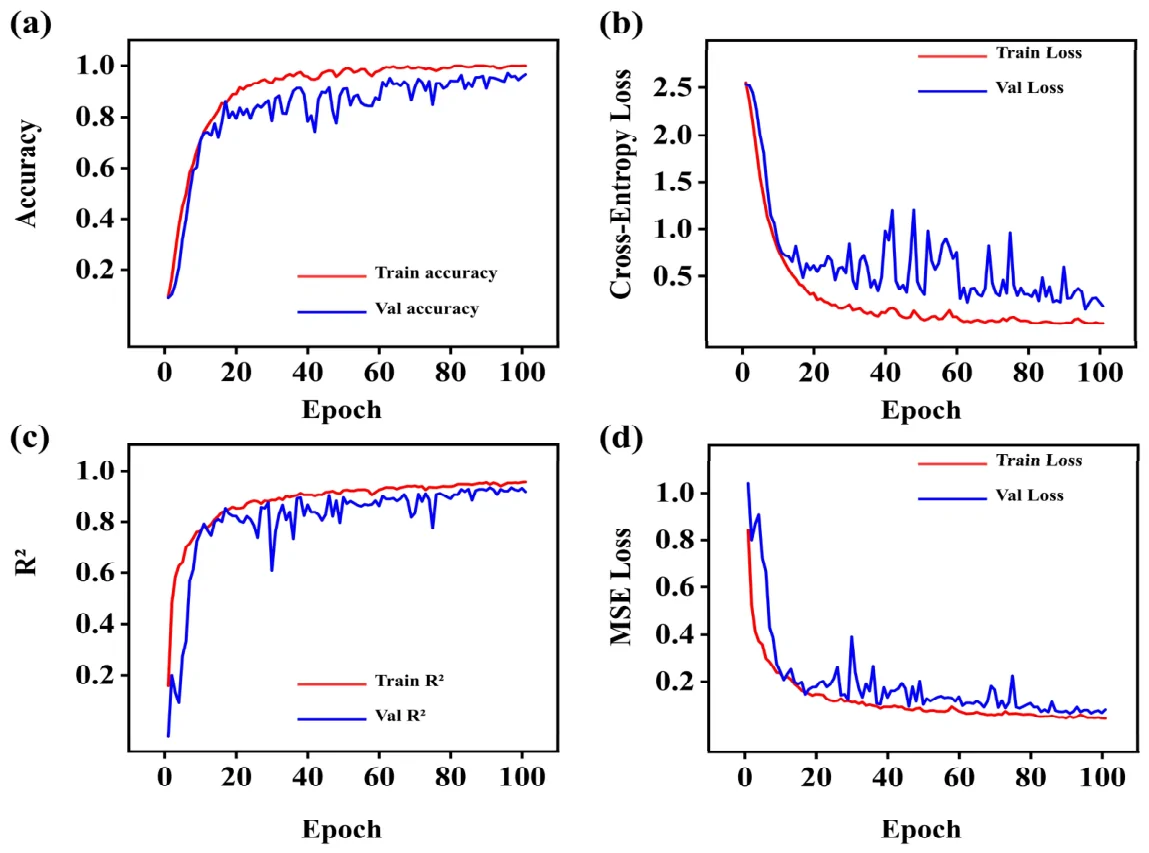
Model training curves. (**a**) Classification Accuracy. (**b**) Cross-entropy loss. (**c**) R^2^. (**d**) Mean squared error loss.

The gas classification loss Loss_cls_ and concentration-regression loss Loss_reg_ were calculated using cross-entropy and mean squared error, respectively. Their learnable log-variance parameters s_cls_ and s_reg_ were used to adaptively balance the two tasks during training. During training, the task-loss weights were adaptively adjusted to reduce the influence of scale differences between the classification and regression losses. Figure 6 presents the training and validation curves for classification Accuracy, cross-entropy loss, regression R^2^, and mean squared error to evaluate model convergence and stability.

Figure 6 shows that the classification accuracy and regression R^2^ increased rapidly during the early training stage, while the cross-entropy loss and MSE decreased and gradually stabilized. Although the maximum number of training epochs was set to 200, early stopping was applied independently to each fold, resulting in training durations of 145, 195, 101, 133, and 200 epochs for Folds 1–5, respectively. Since Fold 3 had the shortest training duration, the first 101 epochs were used to calculate the five-fold averaged learning curves shown in Figure 6, ensuring that all five folds contributed equally to the averaged results at each epoch. Local fluctuations occurred in the validation regression curves, possibly due to large prediction errors for a few samples and adjustments of shared parameters during multitask training. As MSE is sensitive to large errors, a few inaccurate predictions may amplify these fluctuations. Overall, the averaged learning curves showed no sustained deterioration in validation performance within the displayed interval, suggesting good convergence and training stability without obvious signs of overfitting. However, these averaged curves alone cannot fully establish the absence of overfitting in every individual fold. Moreover, the simultaneous convergence of the classification and regression tasks suggests that uncertainty weighting helps balance their contributions during optimization, supporting the effectiveness of the proposed model in jointly learning qualitative VOC identification and quantitative concentration prediction.

### 3.4. Comparative Experiments

**Figure 7:**
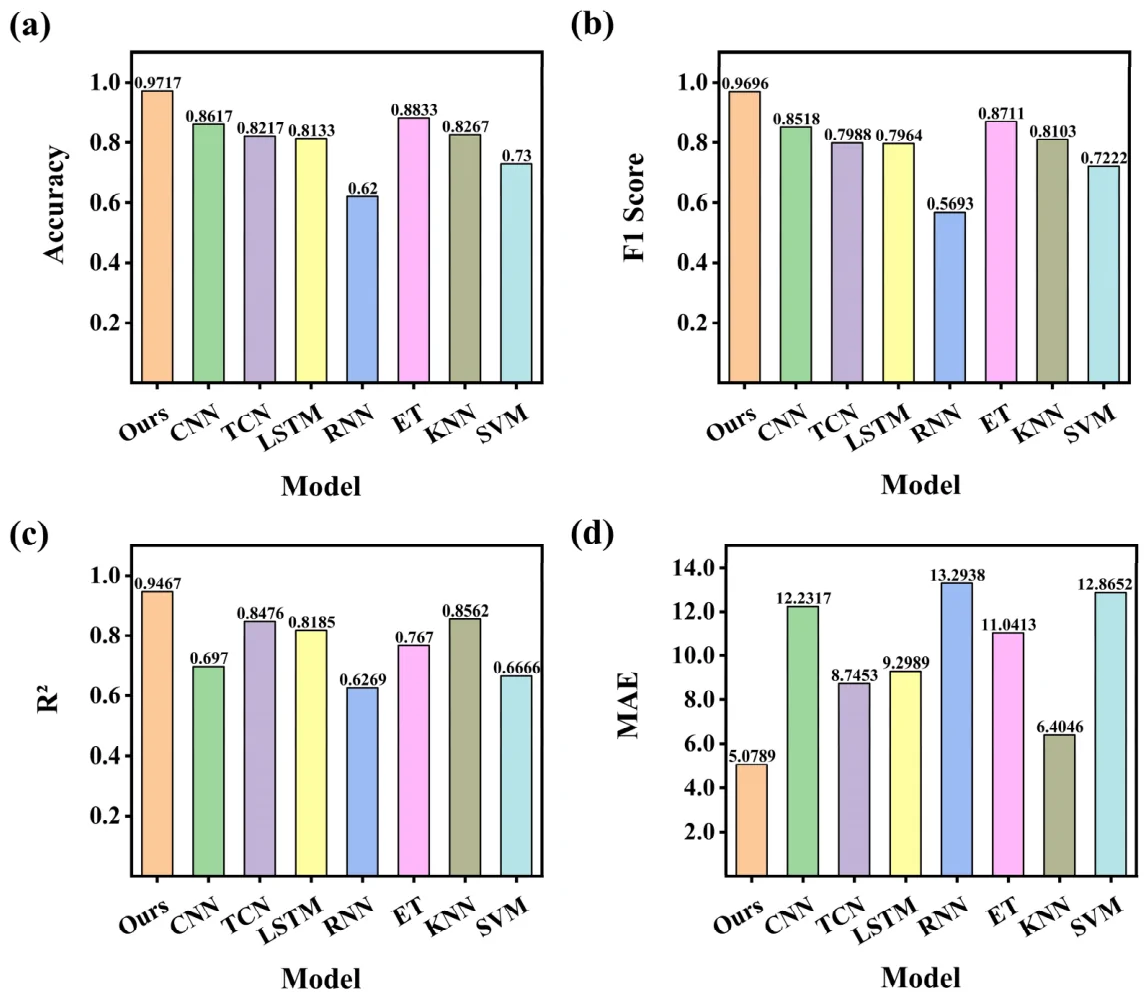
Performance comparison of eight models under five-fold cross-validation. (**a**) Gas identification Accuracy, (**b**) F1-score, (**c**) R^2^ for concentration prediction, and (**d**) MAE for concentration prediction.

To evaluate the proposed model, comparative experiments were conducted using machine-learning models (ET, KNN, and SVM) and deep-learning models (CNN, TCN, LSTM, and RNN). All models employed identical file-level five-fold partitions, with 480 training samples and 120 validation samples per fold, ensuring that samples and overlapping patches from the same source file were not distributed across the training and validation sets. Model hyperparameters were selected based on relevant previous studies and preliminary experiments and kept fixed during the final evaluation. All models employed the same channel-wise Min–Max normalization, with normalization parameters calculated exclusively from the training data of each fold and applied to the corresponding validation data. Model-specific input reshaping was performed where necessary. The comparison results are presented in Figure 7, with detailed metrics provided in Table 5.

The experimental results demonstrate that the proposed model achieves superior overall performance across all evaluation metrics for both gas identification and concentration prediction.

### 3.5. Ablation Experiments

**Figure 8:**
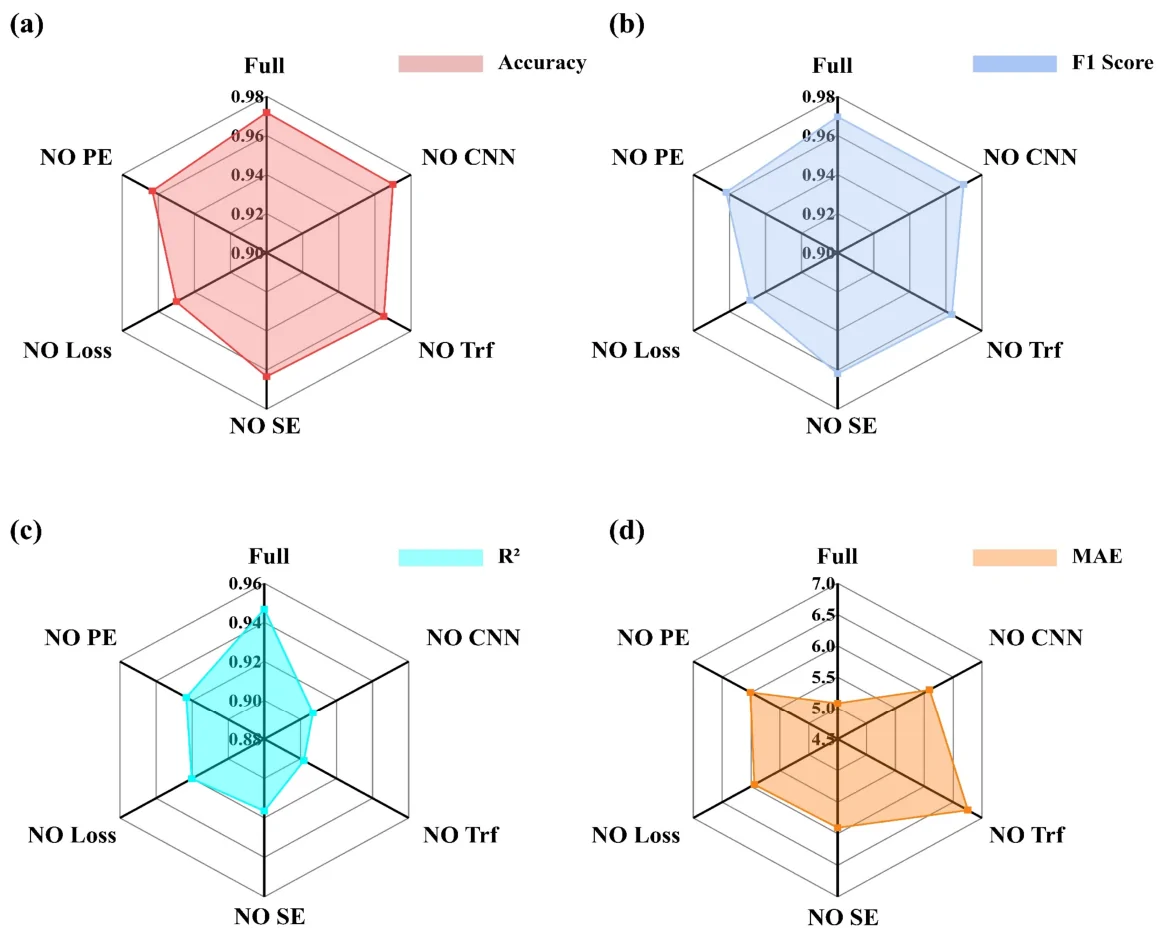
Performance radar charts of different ablated model variants. (**a**) Accuracy, (**b**) F1-score, (**c**) R^2^, and (**d**) MAE.

To evaluate the effectiveness of the key model components, ablation experiments were conducted on the CNN-based local feature encoder, Transformer-based global temporal encoder, task-specific SE attention, multitask loss weighting, and positional embedding. The performance comparison is shown in Figure 8, with detailed results provided in Table 6. The experimental settings are described below.

1. No CNN: The CNN-based local feature encoder was removed to evaluate its contribution to local dynamic feature extraction within response patches and its influence on classification and regression performance.
2. No Trf: The Transformer-based global temporal encoder was removed to evaluate its role in modeling long-range temporal dependencies among response patches.
3. No SE: The task-specific SE attention modules were removed to evaluate their effectiveness in enhancing task-relevant features for classification and regression.
4. No Loss: The multitask loss-weighting mechanism was removed, and the classification and regression losses were directly summed to evaluate its contribution to task balancing and overall model performance.
5. No PE: The positional embedding module was removed to evaluate the importance of patch position information for learning sequential relationships and global temporal features.
6. Full: The complete CNN–Transformer multitask learning model was used for comparison with the ablated variants.

As shown in Table 6 and Figure 8, the full model achieved the best overall performance. Removing the CNN reduced Accuracy by only 0.17 percentage points and left the F1-score nearly unchanged, but increased the MAE from 5.0789 to 6.0918 ppm and reduced R^2^ by 0.0397. This result highlights the contribution of the CNN to extracting concentration-related local dynamic features. Removing the Transformer reduced Accuracy and F1-score by 0.67 and 0.63 percentage points, increased the MAE to 6.7554 ppm, and reduced R^2^ by 0.0448, demonstrating its role in modeling global temporal dependencies among patches. Removing SE reduced Accuracy and F1-score by 0.84 and 0.78 percentage points, increased the MAE to 5.9061 ppm, and reduced R^2^ by 0.0302, indicating that task-specific SE enhances task-relevant features. Without loss weighting, Accuracy and F1-score decreased by 2.17 and 2.11 percentage points, the MAE increased to 5.9430 ppm, and R^2^ decreased by 0.0265, confirming its contribution to balancing classification and regression. Removing positional embedding reduced Accuracy and F1-score by 0.84 and 0.78 percentage points, increased the MAE to 6.0122 ppm, and reduced R^2^ by 0.0235, demonstrating the importance of patch order information. Overall, all components contributed positively, and the full model achieved the best balance between VOC identification and concentration prediction.

### 3.6. Visualization of SE Feature Weights

**Figure 9:**
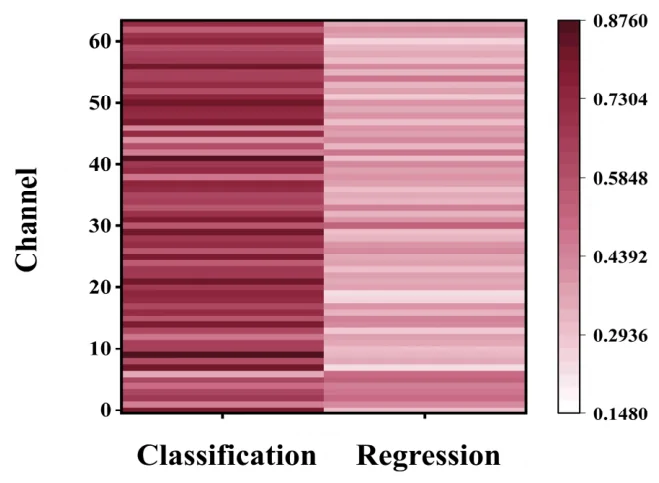
Heatmap of Task-Specific SE Attention Weights.

To analyze the role of task-specific SE attention, Figure 9 visualizes the feature weights of the classification and regression branches. The horizontal axis represents the task branches, while the vertical axis represents the feature channels. Darker colors indicate higher attention weights for the corresponding channels.

Figure 9 shows clear differences in the SE weight distributions of the classification and regression branches. The classification branch assigns relatively high weights to a broader range of feature channels, indicating that gas identification requires multiple discriminative features. In contrast, the high weights in the regression branch are concentrated in fewer channels, which may be more closely related to response intensity and concentration variation. These results demonstrate that task-specific SE attention adaptively recalibrates shared features according to each task objective, thereby enhancing task-relevant feature representations.

## 4. Conclusions

This study proposes a CNN–Transformer multitask learning model based on response-patch sequences for VOC identification and concentration prediction. By comparing different response-patch scales, this study identifies a patch length and stride of 75 and 37 as the optimal configuration, enabling the joint modeling of local dynamic features and global temporal information. Under strict file-level five-fold cross-validation, the model achieved an accuracy of 0.9717 and an F1-score of 0.9696 for gas identification, together with an MAE of 5.0789 ppm and an R^2^ of 0.9467 for concentration prediction, demonstrating favorable performance for both qualitative identification and quantitative prediction. Compared with conventional machine-learning methods and representative temporal deep-learning models, the proposed model achieved superior overall performance. Ablation experiments further confirmed the effectiveness of CNN-based local feature encoding, Transformer-based global temporal modeling, task-specific SE attention, positional embeddings, and multitask loss weighting. These results demonstrate that multitask learning based on response-patch sequences can effectively exploit the dynamic response information of gas-sensor arrays, providing a feasible approach for integrated VOC identification and concentration prediction. However, several limitations remain. The model was evaluated under relatively stable environmental conditions and a fixed ascending concentration sequence, without systematic assessment of environmental variations, incomplete sensor recovery, or exposure-history effects. Furthermore, its generalization to unseen concentration levels and independent external test data remains unverified, and some concentration-dependent prediction bias was observed, particularly at lower concentrations. These limitations reflect the scope of the available experimental data and evaluation protocol. Future work will focus on improving model robustness and concentration prediction accuracy through broader experimental conditions, alternative exposure sequences, and independent testing.

## Figures and Tables

**Table 1:** Information on the Gas Sensors.

Sensor Model	Sensor ID	Nominal Target Gases
MQ-2	Sensor 1	Propane, hydrogen, and methane
MQ-3	Sensor 2	Ethanol
MQ-4	Sensor 3	Methane
MQ-5	Sensor 4	Butane, propane, methane
MQ-6	Sensor 5	Propane, butane
MQ-7	Sensor 6	Carbon monoxide
MQ-8	Sensor 7	Hydrogen
MQ-9	Sensor 8	Carbon monoxide and methane

**Table 2:** Data Preprocessing and Model Hyperparameter Settings.

Parameter	Value
OPTIMIZER	Adam
K_FOLDS	5
NUM_EPOCHS	200
BATCH_SIZE	8
SEED	42
LEARNING_RATE	0.0005
PATIENCE	35
LR_FACTOR	0.5
LR_PATIENCE	12
MIN_LR	0.000001
REDUCTION	8

**Table 3:** Model Performance with Different Response-Patch Lengths.

Segment Setting (Length/Stride)	Segment	Accuracy	F1-Score	MAE	R^2^	Params
55/27	38	0.9617	0.9601	5.7746	0.9276	71,517
75/37	28	0.9717	0.9696	5.0789	0.9467	70,877
95/47	22	0.9650	0.9636	5.5032	0.9315	70,493
115/57	18	0.9683	0.9661	5.6159	0.9169	70,237
135/67	15	0.9617	0.9602	5.2433	0.9414	70,045
155/77	13	0.9583	0.9570	5.1890	0.9336	69,917
175/87	12	0.9650	0.9639	5.5688	0.9342	69,853
195/97	10	0.9583	0.9567	5.7986	0.9236	69,725
1050/1050	1	0.9633	0.9622	5.7431	0.9212	69,149

**Table 4:** Five-Fold Cross-Validation Performance (Mean ± Std.).

Accuracy	Std.	F1-Score	Std.	MAE	Std.	R^2^	Std.
0.9717	0.0326	0.9696	0.0366	5.0789	0.9642	0.9467	0.0171

**Table 5:** Classification and Regression Performance of Eight Models.

Model	Accuracy	F1-Score	MAE	R^2^
Ours	0.9717	0.9696	5.0789	0.9467
CNN	0.8617	0.8518	12.2317	0.6970
TCN	0.8217	0.7988	8.7453	0.8476
LSTM	0.8133	0.7964	9.2989	0.8185
RNN	0.6200	0.5693	13.2938	0.6269
ET	0.8833	0.8711	11.0413	0.7670
KNN	0.8267	0.8103	6.4046	0.8562
SVM	0.7300	0.7222	12.8652	0.6666

**Table 6:** Ablation Study Results.

Model	Accuracy	F1	MAE	R^2^
No CNN	0.9700	0.9698	6.0918	0.9070
No Trf	0.9650	0.9633	6.7554	0.9019
No SE	0.9633	0.9618	5.9061	0.9165
No loss	0.9500	0.9485	5.9430	0.9202
No PE	0.9633	0.9618	6.0122	0.9232
Full	0.9717	0.9696	5.0789	0.9467

## Data Availability

The data supporting the findings of this study are available from the corresponding author upon reasonable request.

## Supplementary Materials

The following supporting information can be downloaded at https://www.mdpi.com/article/10.3390/s26216961/s1. Figure S1: Electronic-nose system and data acquisition procedure. (a) Experimental setup; (b) VOC exposure and recovery sequence; (c) Schematic sensor response and recovery process; Figure S2: Time–response curves of individual sensors to 12 VOCs at 10–100 ppm. (a–h) Responses of Sensors 1–8, respectively; (i) Color legend for the 12 VOCs.

## References

[1] Khatib M., Haick H. (2022). Sensors for Volatile Organic Compounds. ACS Nano.

[2] Li Y., Wang Z., Zhao T., Li H., Jiang J., Ye J. (2024). Electronic nose for the detection and discrimination of volatile organic compounds: Application, challenges, and perspectives. TrAC Trends Anal. Chem..

[3] Karakaya D., Ulucan O., Turkan M. (2020). Electronic Nose and Its Applications: A Survey. Int. J. Autom. Comput..

[4] Ye Z., Liu Y., Li Q. (2021). Recent Progress in Smart Electronic Nose Technologies Enabled with Machine Learning Methods. Sensors.

[5] Tan Y., Chen Y., Zhao Y., Liu M., Wang Z., Du L., Wu C., Xu X. (2025). Recent advances in signal processing algorithms for electronic noses. Talanta.

[6] Bakiler H., Güney S. (2021). Estimation of Concentration Values of Different Gases Based on Long Short-Term Memory by Using Electronic Nose. Biomed. Signal Process. Control.

[7] Zeng L., Xu Y., Ni S., Xu M., Jia P. (2023). A mixed gas concentration regression prediction method for electronic nose based on two-channel TCN. Sens. Actuators B Chem..

[8] Mao G., Zhang Y., Xu Y., Li X., Xu M., Zhang Y., Jia P. (2023). An electronic nose for harmful gas early detection based on a hybrid deep learning method H-CRNN. Microchem. J..

[9] Stefanone A., Meacci L., Rossoni M., Colombo G. (2025). Transformer-based odor recognition on E-nose platforms. Results Eng..

[10] Liu H., Li Q., Gu Y. (2020). A multi-task learning framework for gas detection and concentration estimation. Neurocomputing.

[11] Wang T., Zhang H., Wu Y., Jiang W., Chen X., Zeng M., Yang J., Su Y., Hu N., Yang Z. (2022). Target discrimination, concentration prediction, and status judgment of electronic nose system based on large-scale measurement and multi-task deep learning. Sens. Actuators B Chem..

[12] Ni W., Wang T., Wu Y., Chen X., Cai W., Zeng M., Yang J., Hu N., Yang Z. (2024). Classification and Concentration Predictions of Volatile Organic Compounds Using an Electronic Nose Based on XGBoost-Random Forest Algorithms. IEEE Sens. J..

[13] Feng Z., Wu F., Zhao L. (2024). A Multitask Electronic Nose Data Processing Model Based on Transformer Encoder. IEEE Sens. J..

[14] Jiang M., Li N., Li M., Wang Z., Tian Y., Peng K., Sheng H., Li H., Li Q. (2024). E-Nose: Time–Frequency Attention Convolutional Neural Network for Gas Classification and Concentration Prediction. Sensors.

[15] Ni W., Wang T., Wu Y., Liu X., Li Z., Yang R., Zhang K., Yang J., Zeng M., Hu N. (2024). Multi-task deep learning model for quantitative volatile organic compounds analysis by feature fusion of electronic nose sensing. Sens. Actuators B Chem..

[16] Wang X., Zhao W., Ma R., Zhuo J., Zeng Y., Wu P., Chu J. (2024). A novel high accuracy fast gas detection algorithm based on multi-task learning. Measurement.

[17] Dai Y., Xiong Y., Lin H., Li Y., Feng Y., Luo W., Zhong X. (2024). A Multitask Learning-Based Model for Gas Classification and Concentration Prediction. IEEE Sens. J..

[18] Mei H., Yang R., Peng J., Meng K., Wang T., Wang L. (2025). Research on Binary Mixed VOCs Gas Identification Method Based on Multi-Task Learning. Sensors.

[19] Li L., Zhao Y., Tong B., Cao F., Zhang R., You Y., Zhu H. (2025). Rapid Classification and Concentration Prediction of VOC Gaseous Mixtures Using Sensor Array With Multitask Learning. IEEE Sens. J..

[20] Wu F., Ma R., Li Y., Li F., Duan S., Peng X. (2024). A novel electronic nose classification prediction method based on TETCN. Sens. Actuators B Chem..

[21] Yang Y., Wang Z., Ren Z., Chen Y., Guo X., Yin C., Shi Y. (2025). MCCT-Net: A hybrid neural network for improving the detection performance of electronic nose system. Sens. Actuators B Chem..

[22] Jia P., Chen Z., Mao G., Zhang Y., Liu J., Xu M. (2025). Gas concentration prediction based on temporal attention mechanism in temporal convolutional networks. Sens. Actuators B Chem..

[23] Chen X., Xia X., Zhuo J., Wu P., Peng X., Chu J. (2026). SADDCN-HFF: A lightweight hybrid network electronic nose system for mixed gas concentration prediction. Sens. Actuators B Chem..

[24] Wang B., Wu K., Tang J., Liu H. (2026). MT-TERN: A multi-task network based on Transformer-ResNet1D for gas classification and concentration prediction. Results Eng..

[25] Fu C., Zhang K., Guan H., Deng S., Sun Y., Ding Y., Wang J., Liu J. (2024). Progressive prediction algorithm by multi-interval data sampling in multi-task learning for real-time gas identification. Sens. Actuators B Chem..

[26] Karpontinis D., Alexandridis G. (2026). Patch-Based Transformers for Long-Term Energy Consumption Forecasting. Int. J. Artif. Intell. Tools.

[27] Kim T., Kim Y., Cho W., Kwak J.-H., Cho J., Pyeon Y., Kim J.J., Shin H. (2024). Ultralow-Power Single-Sensor-Based E-Nose System Powered by Duty Cycling and Deep Learning for Real-Time Gas Identification. ACS Sens..

[28] Mei H., Peng J., Wang T., Zhang B., Xuan F. (2026). Gas Identification Driven by Dynamic Response Fragments and Multi-Task Learning. Anal. Chem..

[29] Ma X., Wu F., Yue J., Feng P., Peng X., Chu J. (2024). MSE-TCN: Multi-scale temporal convolutional network with channel attention for open-set gas classification. Microchem. J..

[30] Vandenhende S., Georgoulis S., Van Gansbeke W., Proesmans M., Dai D., Van Gool L. (2022). Multi-Task Learning for Dense Prediction Tasks: A Survey. IEEE Trans. Pattern Anal. Mach. Intell..

[31] Zeng Y., Liu Z., Liu Z., Peng X., Cui H., Yan J., Duan S., Wang L., Chu J. (2025). A lightweight gas classification and concentration prediction method based on PTQ-CNN by using an electronic nose system. Sens. Actuators A Phys..

[32] Mei H., Peng J., Wang T., Zhang B., Xuan F., Wang L., Zeng M., Yang Z. (2025). Smart VOCs recognition system based on single gas sensor and multi-task deep learning model. Sens. Actuators B Chem..

[33] Yang J., Hu X., Feng L., Liu Z., Murtazt A., Qin W., Zhou M., Liu J., Bi Y., Qian J. (2024). AI-Enabled Portable E-Nose Regression Predicting Harmful Molecules in a Gas Mixture. ACS Sens..

[34] Föllmer B., Biavati F., Wald C., Stober S., Ma J., Dewey M., Samek W. (2022). Active multitask learning with uncertainty-weighted loss for coronary calcium scoring. Med. Phys..

