# Supplementary material for: Response-Patch CNN–Transformer Multitask Learning Model for VOCs Identification Using Electronic Nose
Source: Sensors (Basel). 2026 Nov 4;26(21):6961. doi: 10.3390/s26216961 (PMC13766527; doi:10.3390/s26216961)
Supplement: Supplementary file 1 [file sensors-26-06961-s001.zip › sensors-4648122-supplementary.pdf]

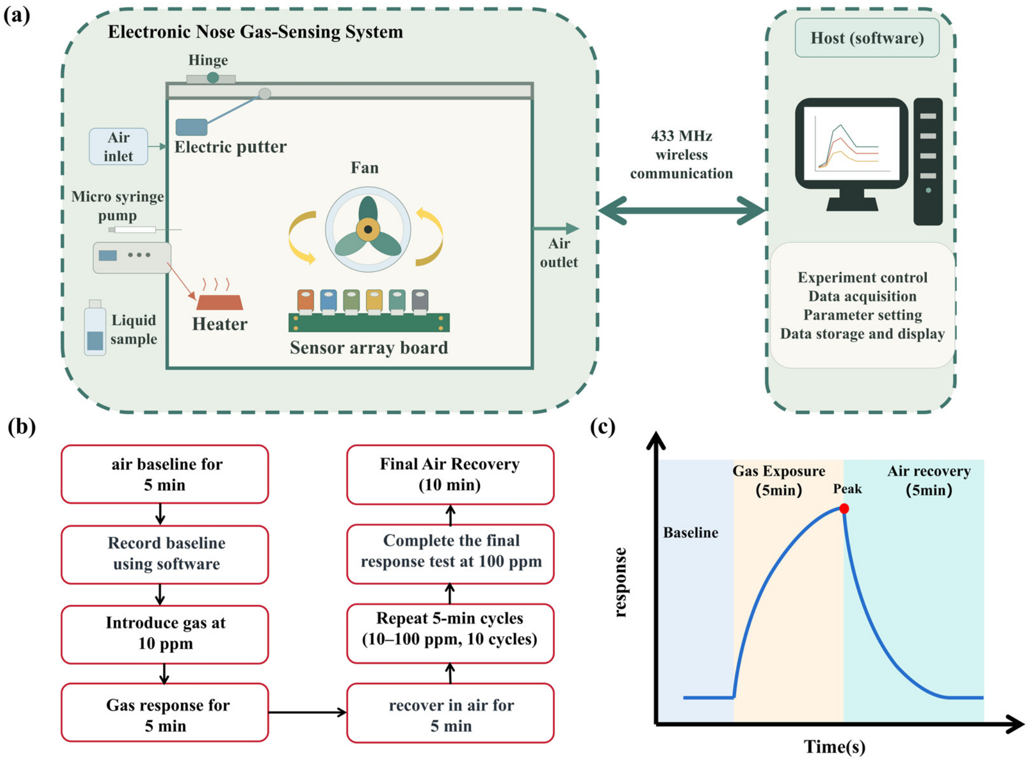

Figure S1. Electronic-nose system and data acquisition procedure. (a) Experimental setup; (b) VOC exposure and recovery sequence; (c) Schematic sensor response and recovery process.

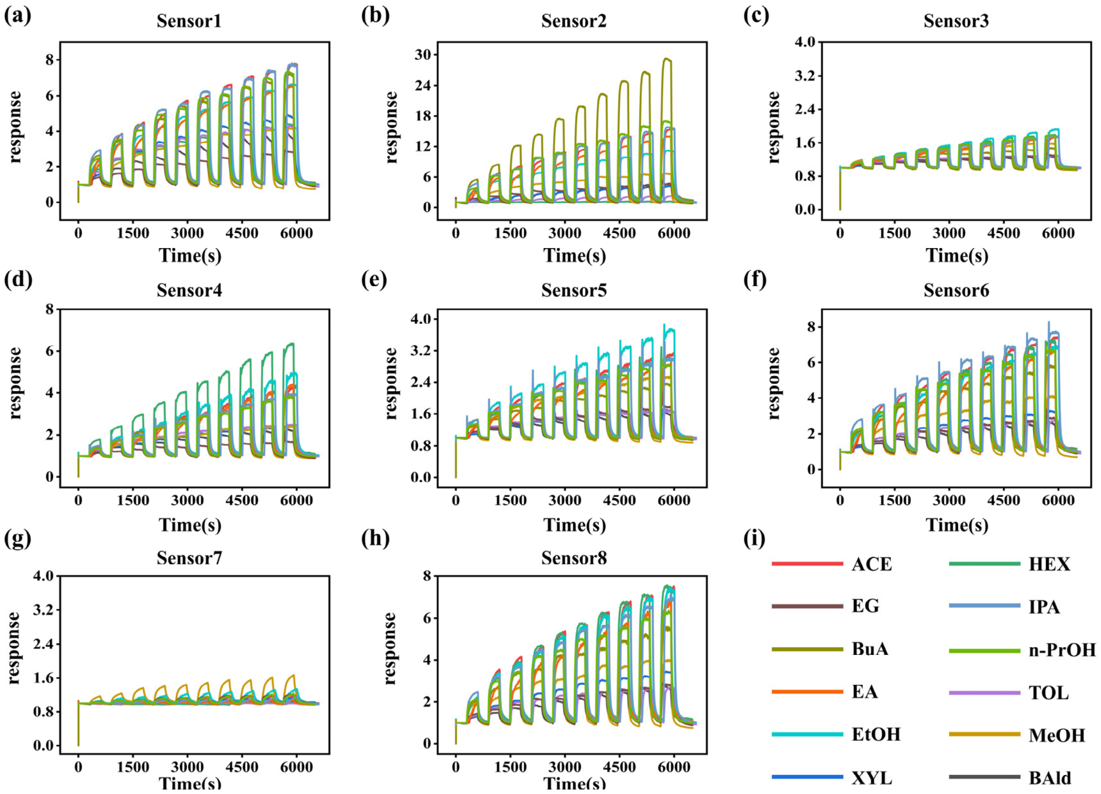

Figure S2. Time–response curves of individual sensors to 12 VOCs at 10–100 ppm. (a–h) Responses of Sensors 1–8, respectively; (i) Color legend for the 12 VOCs.
